# Exploring the Climate Change, Migration and Conflict Nexus

**DOI:** 10.3390/ijerph13040443

**Published:** 2016-04-22

**Authors:** Kate Burrows, Patrick L. Kinney

**Affiliations:** Mailman School of Public Health, Columbia University, New York, NY 10024, USA; plk3@cumc.columbia.edu

**Keywords:** climate change, migration, conflict

## Abstract

The potential link between climate change, migration, and conflict has been widely discussed and is increasingly viewed by policy makers as a security issue. However, considerable uncertainty remains regarding the role that climate variability and change play among the many drivers of migration and conflict. The overall objective of this paper is to explore the potential pathways linking climate change, migration and increased risk of conflict. We review the existing literature surrounding this issue and break the problem into two components: the links between climate change and migration, and those between migration and conflict. We found a large range of views regarding the importance of climate change as a driver for increasing rates of migration and subsequently of conflict. We argue that future research should focus not only on the climate-migration-conflict pathway but also work to understand the other pathways by which climate variability and change might exacerbate conflict. We conclude by proposing five questions to help guide future research on the link between climate change, migration, and conflict.

## 1. Introduction

The potential link between climate change, migration, and conflict has been discussed in academic literature for several decades. Increasingly, policy makers and media sources recognize climate as a security issue. However, despite this growing concern and focus on climate change and conflict, uncertainty remains regarding the pathways linking climate change to migration to conflict. This uncertainty is brought about, in part, by the inherent complexity of climate change projections. It is furthered by the challenges of accurately projecting population growth and movements, identifying the outbreak of conflict, and determining the significance of climate and migration as drivers of conflict relative to other stabilizing or destabilizing forces. Despite these challenges and inherent uncertainty, the potential consequences are so severe that it is essential that further research be conducted to better understand the possible linkages between climate change, migration, and conflict. This paper reviews the available literature on the climate-migration-conflict nexus by firstly, examining the link between climate change and migration, and secondly, the link between migration and conflict. Additionally, we have summarized some of the key case studies in recent literature (since 2000) in Table 1. This table provides a sample of the types of studies that are being conducted on climate extremes, migration, and conflict. Finally, we propose a serious of questions to help identify local contexts in which climate-migration might increase risk of conflict. 

This paper will discuss both short and long term migration and some of the ways in which climate extremes impact rates of both types of movement. We use the word conflict broadly to encompass violence at the interpersonal, intergroup, and international levels. While some of the literature focuses specifically on one type of violence, such as civil conflict [14], a wider definition of conflict is also common [15,16,17]. This paper is focused on conflict that can occur in receiving areas upon or shortly after migration. It is possible that some conflict might occur during migration, or well after migration has already occurred; however, this is not well documented in the literature. Further disaggregation of scales of violence as they relate to specific types of migration will be an important focus for future research, but is not within the scope of this paper.

**Figure 1:**
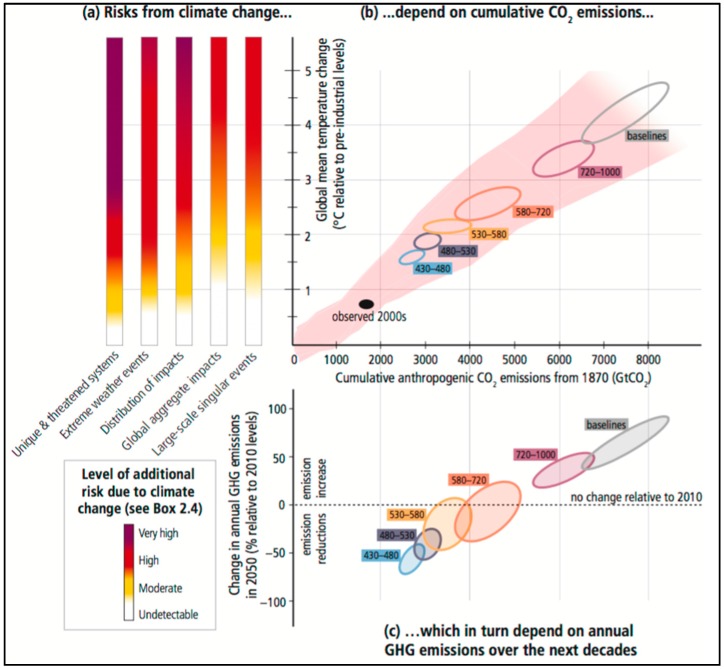
The relationship between risks from climate change, temperature increase and carbon dioxide emissions, and changes in annual greenhouse gas emissions. Reproduced from the Intergovernmental Panel on Climate Change (IPCC) Fifth Assessment Report [18]. (**a**) shows the level of additional risk due to climate change for five different threats. The risk per threat is scaled from “Very high” to “Undetectable” (see color legend below (**a**)) for each increase in global mean temperature; (**b**) shows the relationship between cumulative greenhouse gas emissions (in GtCO_2_) and changes in global mean temperature. The ellipses represent CO_2_ emissions under the different IPCC scenario categories; (**c**) links cumulative CO_2_ emissions to changes in annual greenhouse gas emissions by 2050.

The most recent Intergovernmental Panel on Climate Change (IPCC) Assessment Report states that it is extremely likely that anthropogenic factors are the cause of more than half of the observed increase in the average global surface temperature over the last 60 years [18]. We have already observed changes in extreme weather events and it is very likely that we can expect to see increases in the length and severity of heat waves and extreme storm events in the future [18]. Of particular relevance for livelihoods will be changes in patterns of seasonal rainfall, which among other things will lead to shifts in regional suitability for agriculture. While such changes are inevitable, climate models do not agree well on where and when such changes will occur. It is also important to note that projections of future climate depend on assumptions of greenhouse gas emissions, and these assumptions also vary greatly depending on socio-economic development and policy changes. Figure 1 (reproduced below) demonstrates this uncertainty.

The potential for global environmental change to result in conflict has been discussed since the 1980s [15,19]. There is increasing discourse about the “securitization” of climate change, or the process by which climate change has been reframed from a purely environmental issue to a security threat in order to increase awareness and inspire action [20]. Some scholars suggest that this process is still ongoing [20], while others argue that it has already failed [21]. A number of different pathways between climate change and conflict have been proposed and discussed. These include declines in agricultural productivity leading to food shortages, water scarcity, and competition for mineral resources (including gas and oil) [22]. Among the most frequently cited link between climate change and conflict is the potential for increased migration [15].

The climate-migration-conflict pathway has received increased focus from policy makers and the media [23]. A popular view has emerged in these circles that climate change will lead to a dramatic increase in movement of people away from impacted areas and will result in increased conflict with populations in areas receiving migrants. Despite (and perhaps in response to) the fact that this issue is viewed as relatively linear and even deterministic in the media, scholars have been increasingly cautious when discussing the climate-migration-conflict pathway. In fact, there remains no real consensus about whether or not this pathway exists, whether it can be considered causal, and how future research could fill critical knowledge gaps.

## 2. Climate Change-Induced Migration

Climate variability and change bring the risk of serious negative impacts on environmental and human systems, including extreme events such as drought, heat waves, floods, storms, and wildfires and slower onset impacts such as changing rainfall patterns, sea-level rise, increased salinization, decreased soil fertility, and others [18]. These events could lead to population displacement as migrants relocate due to the damage or loss of land and property [24,25]. However, there is disagreement surrounding the specific ways in which climate change will impact migration and how significant climate change can be as a determining factor in the decision to migrate. 

Because climate change is by definition a long-term process, and due to the uncertain attribution of extreme events to climate change, we lack the data and tools to directly document impacts of climate change per se on migration. Instead, most work in this area examines impacts of shorter-term climate variability (*i.e.*, extremes) to inform our understanding of how longer-term climate change may impact population health (*i.e*., flooding as a proxy for sea-level rise). This assumption is implicit in most climate change and migration literature. It has been argued that if we wait for climate-change induced impacts to be clearly evident, it may be far too late to take action to prevent them [26].

### 2.1. Estimating the Number of Environmental Migrants

Migration includes both short-term and long-term movements [27]. Published estimates of the numbers of people who may be displaced by climate change related phenomena by 2050 range from 50 million to 1 billion people [28,29]. Jacobson put forward the low end of the range (50 million environmental refugees) as an estimate of populations “at-risk” of a 1 m sea level rise [30]. The most commonly cited number, 200 million, is based on projections conducted by Norman Myers in 1995 and 2000 [31,32,33]. This projection has not been rigorously tested, but is generally agreed upon as consistent with conservative estimates of climate impacts [28,34]. However, Myers himself has admitted to uncertainty in his estimate and that necessary extrapolations were made due to a lack of available data [34]. The high-end estimate, 1 billion people displaced by 2050, comes from a Christian Aid report [29]. This report breaks the estimate into 250 million people displaced by climate change related events, 50 million people displaced by natural disasters, 50 million people displaced by conflict, 5 million people fleeing their countries and being accepted as refugees, and the majority, 645 million people displaced by development projects [29]. Use of this number by the media and in academic literature is sometimes considered alarmist [35,36,37]. The large range in these estimates illustrates the enormous uncertainty surrounding the causal impacts of climate change on migration.

### 2.2. The Environment as a Contextual Determinant of Migration

**Figure 2:**
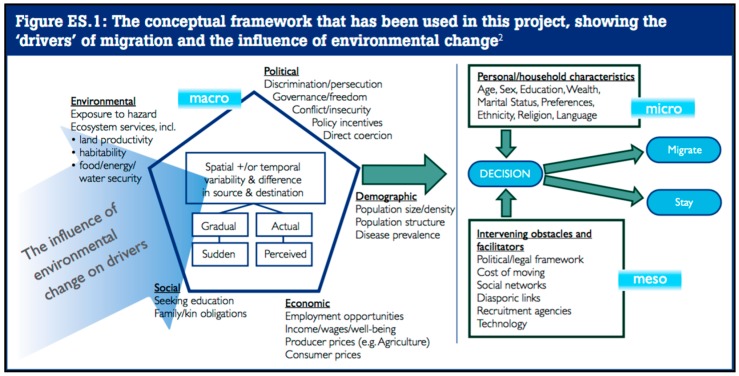
Conceptual model for understanding the role of the environment as one of many drivers of migration [30]. This model is reproduced from the UK Foresight Report (2011) and is intended to demonstrate the complexity of the contextual factors that can lead to migration [30]. (Contains public sector information licensed under the Open Government Licence v3.0.) This paper focuses on the role of climate change as an environmental driver, but we also discuss the relative roles of political and economic factors as stabilizing and/or destabilizing forces.

A great deal of the academic literature that addresses climate-migration is based on traditional migration theory, which puts forward that drivers of migration can be broken into five broad categories. These include factors that promote out-migration (including the environment, political instability, conflict, lack of economic opportunity, *etc.*); factors that draw in-migrants (including economic opportunity, demand for resources, political stability, *etc.*); “network” factors, which facilitate or hinder the move between the two places (including family ties, ease of transport, legality of migration, *etc.*); national policies that hinder or encourage movement; and the personal goals or motivations of the migrant [23,38]. Fundamental to the theory of climate-migration is the postulate that changes in the environment can serve as a potential contextual driver that will encourage individuals to leave their homes as environmental stresses make living in one place no longer feasible or desirable [34]. However, these factors and their interactions are highly complex, poorly understood, and context specific. Because of the variety of different reasons for migration, it is often difficult to isolate any one driver as necessary or sufficient. For example, land degradation might negatively impact economic opportunity and influence out-migration—in this case, is the driver the environment, or is it lack of economic opportunity? This is one of the key complexities in climate-migration research. A fairly complex model was developed for the 2011 UK Foresight report (2011) that describes how environmental drivers can (along with many other factors) lead to migration [30]. This graphic is reproduced below (Figure 2) in order to illustrate the challenge of determining the relative role of any one of these factors. 

Some have suggested that the climate-migration literature has put too much weight on the significance of the environment as a driving factor of out-migration. For example, Brzoska and Frohlich (2015) argue that the emphasis on environmental factors as drivers of migration is in fact selective and inconsistent with migration literature [15]. The implications of this are substantial: if the environment is not the significant a contextual factor that some climate-migration literature assumes it to be, the potential number of those displaced by climate change might be markedly lower than current estimates. In contrast, Reuveny (2007) argues that areas that depend more on the environment (*i.e*., depend on agriculture for livelihood) will see more environmental migration [1]. This highlights the importance of local context in determining how important environmental factors may be as drivers of migration. 

### 2.3. Type of Migration

Migration can be either internal or international and either temporary or permanent [39]. Most migration after extreme weather events is temporary [39]. However, as will be discussed in Section 2.5, different disasters impact rates of temporary and permanent migration differently and are intrinsically tied to other factors such as economic status. 

The choice of destination is affected largely by distance from the place of origin (most migration occurs over short distances), availability of jobs and housing, family and social networks, and immigration policies in the new location [40]. For example, environmental migrants leaving rural areas due to natural disasters may try to find work in local cities [34]. Furthermore, environmental migrants are more likely to stay within their own countries than they are to migrate abroad [41,42,43]. In both developed and developing countries, migrants after extreme natural events have been observed to remain within national borders and close to their place of origin. For example, in Florida after Hurricane Andrew, 76% of 353,300 displaced residents from Dade County remained within the county, 18% moved within the state, and 6% moved out-of-state ([44], p. 272). Similarly, a study in India of the Ghaghara floodplain found that the majority of permanent migrants settled just outside the flood zone [45]. After the Indian Ocean Tsunami in 2004, migration to nearby urban areas was observed in Sri Lanka, Thailand, and Indonesia [46]. These findings suggest that migration as an adaptive response to natural hazards most often occurs within a very small radius of the migrant’s place of origin, and may tend towards urban areas. However, despite the fact that the majority of migration after natural disasters is internal, most research to-date has focused on international migration [39]. This highlights the issue of localized migration as one priority for future research. 

### 2.4. Migration as Adaptation

Most of the available estimates for the numbers of people who may be displaced by climate change are rooted in the simplistic view that migrants move only as a “last-resort” in response to unbearable circumstances. However, migration is increasingly being recognized as a viable coping or adaptation strategy [37,47]. In fact, in many cases it is those who do not migrate (either because they cannot or will not) who are at the greatest risk [48]. Individuals who do migrate, for example, may send remittances to their families, thus strengthening the home community’s resilience to climate extremes [49]. Migrants may also transfer knowledge and technology to their community of origin, which can reduce vulnerability at home [30]. While in the host community, migrants will often form networks among themselves [49]. These migrant-networks may also integrate into the host community, creating a link between home and host [49]. This link can then facilitate future migration and movement of ideas, money, and resources [30]. The roles of social capital and social integration in limiting conflict in the host community will be discussed later.

If migration can be viewed in part as an adaptive response to changing circumstances (e.g., socioeconomic, environmental, or cultural), it requires a shift to understanding why migrants make the decision to move [37,47]. The UN Framework Convention on Climate Change incudes migration as a form of adaptation, and some governments are considering resettlement as a potential strategy [27]. However, it should be noted that adaptation-related relocation plans have often been met with resistance due to a fear of cultural loss or increased psychological stress associated with moving [27]. 

### 2.5. Evidence of Climate Extremes and Resource Scarcity Impacting Migration

Most studies that examine the link between climate change and migration use climate variability as a proxy to climate change, as has been discussed above. While not diminishing the importance of these studies, it does complicate their interpretation, particularly when using these studies to inform policy decisions. It is also important to note that environmental change may both increase and decrease rates of migration, and that it is important to consider which types of migration are being altered. The majority of studies on migration as a result of environmental stress are focused on floods (including hurricanes, storm surge, *etc.*) and drought (including soil degradation and impacts on farming). Key findings are discussed below.

Drought has been linked to an increase in both short-term migration (as a way to diversify income) and long-term migration [30,50]. For example, in Western Sudan in the late 1990s, migration to search for wage labor was used as an adaptive strategy to increase economic opportunity during drought [51]. Historically, drought has also been linked to increased permanent migration. Benson, Petersen, and Stein suggest that severe droughts in the 12th and 13th centuries may have impacted migration and abandonment of a number of pre-Columbian Native American villages [52].

Soil degradation, linked to drought as well as unsustainable agricultural practices, has also been tied to both increasing and decreasing rates of overall migration. Gray (2011) noted an increased rate of short-term labor-related migration with increased soil degradation in Kenya, suggesting that migration is used as a common coping strategy to diversify income [2]. However, Gray also found that decreased soil quality slightly reduced rates of overall migration in Uganda [2]. Soil degradation in Uganda reduced the capacity of households to be able to afford to send permanent, non-labor migrants [2]. The participants in Uganda potentially represent a “trapped population”, whose limited capital prohibited migration [30]. Such populations are particularly vulnerable because their limited wealth not only reduces the feasibility of migration but also limits the potential for successful adaptation [30]. The conflicting results of this study demonstrate that environmental change is not linked linearly to migration but rather depends on a range of other drivers that can influence the migration decision (here, income). The role that environmental changes may play as drivers of increased migration requires consideration of the specific local context for decisions about migration. 

Flooding, often caused by extreme storm events, of a driver migration. Mallick and Vogt found that migration rates were much higher in lower socioeconomic segments of Bangladeshi society after Cyclone Aila [5]. This was largely due to a need to diversify income and the inability to rebuild lost or damaged property [5]. This mirrors findings in drought literature that income can be a determining factor in migration during or after periods of environmental stress. However, a review of migration in response to natural disasters that occurred in the United States in the 1920s and 1930s found that flood events were not associated with increases in out-migration [53]. This may be due in part to public efforts at disaster mitigation, including rebuilding after storms and increasing protection in flood-prone areas [53]. This suggests that perhaps governmental support has the capacity to outweigh other factors that might influence migration. Similarly, overall migration was relatively limited after the 2004 Indian Ocean tsunami [37]. It is suggested that this is due in part to rapid humanitarian response. Also in 2004, Hurricane Ivan damaged 89% of housing in Grenada, but within a year almost 60% of housing had been repaired to pre-hurricane levels [54]. This suggests very low levels of long-term migration, due in part to government and donor support for rebuilding [54]. The relative roles of political stability and economic status will be discussed in further detail later. 

Most of the literature to-date on climate and migration has focused on droughts and flooding. Much less research has examined the influence of temperature or fire extremes as migration drivers. In rural Pakistan, heat stress is associated with greater increases in long-term migration than flooding [3]. This may be due to limited emergency relief after heat events compared with response to floods [3]. Forest fires have also been linked to an increased intention to migrate [4]. Most migration after fires is temporary, much like migration after other natural disasters; however, unlike after floods and droughts, researchers have found no difference in intention to migrate between socioeconomic groups after fires [4,30]. Despite these intriguing results, there is little to no available research on the specific mechanisms by which these extremes might impact migration in the context of climate change.

## 3. Linking Migration to Conflict

As we have seen, the link between climate and migration is difficult to isolate. We must turn a similarly critical eye to the second pathway—the potential for migration to increase the risk of conflict. Current literature surrounding migration and conflict increasingly suggests that climate change and climate-related migration will not *cause* conflict independent of other important political and economic factors [14,16,17]. While it is agreed that climate change will not alone cause conflict, it is also acknowledged almost universally that it has the potential to exacerbate or catalyze conflict in conjunction with other factors. However, how important a factor migration may be in terms of causing conflict is open to debate [17]. This section will discuss first the evidence that links climate-migration to increased risk of conflict, and second the counter arguments that suggest that this pathway oversimplifies the relationship between climate-migration and conflict. 

### 3.1. Arguments for Migration as a Catalyst of Conflict

There are a number of examples of non-climate related migration leading to increased tensions and conflict, particularly over international borders. For example, mass migration from Bangladesh into Northern India over the last few decades has resulted in tension and conflict between ethnic groups [55,56]. Intergroup violence has been observed in Western Europe between West African immigrants in France and Indian immigrants in Great Britain [57]. Presently, the Syrian refugee crisis in Europe is causing tension among the member states of the European Union (EU) [58]. In general, larger countries with stronger economies (e.g., Germany and Sweden) have been more successful in meeting EU quotas for distribution of refugees [58]. 

Ethnic tensions between migrants and residents in receiving areas have been discussed as potential causes for conflict. This often arises as a result of the migrant being viewed as the “other”. However, this type of thinking has implications not just in terms of ethnic tensions, but also in terms of related socioeconomic tensions. Migrants often compete for jobs with locals and migrants’ “otherness” may provoke resentment and ultimately conflict. This has been seen, for example, during rural-urban migration where former pastoralists compete for positions in the urban market [59]. “Otherness” may also go beyond the individual level and extend to issues of national identity, particularly if the migration is across international borders [60]. Receiving countries may feel overwhelmed and potentially threatened by the influx of people with different languages and religions. More broadly, it is significant to note that uncertainty about the future is one of the most crucial factors that can lead to violent conflict, and in some ways perceived insecurity is more critical than actual insecurity [16]. As such, even if in reality migrants do not pose a significant threat to political or economic power, the perceived risk may be enough to provoke conflict.

While the above offers a general explanation of how migration may cause conflict, in the context of climate change it is also important to consider that increased resource competition can further exacerbate the potential for migration to lead to conflict. This concept has been described as “neo-Malthusian” and argues that as populations continue to increase, competition for resources will also increase, and that resources will already be scarcer due to climate change [61]. Migration might further exacerbate this competition and could thereby result in conflict as the inequalities increase between those in control of resources and those who do not have access. 

It is important to reiterate that this literature does not imply that climate-migrants will, in isolation, result in increased violence. Rather, climate-migration may (as other migrations have been shown to do in the past) act as an exacerbating factor that can increase risk of violence, particularly in the context of neo-Malthusian resource scarcity. This mirrors the current challenge surrounding climate and migration. The greatest uncertainty regarding conflict is how significant a driver migration will be as a catalyst to conflict. The following section will discuss why current literature is becoming increasingly cautious about overstating the potential impact of climate-migration on conflict.

### 3.2. Complexities of Viewing Migration as a Catalyst of Conflict

One of the simplest, but starkest, counter-arguments to the potential for climate-induced migration to lead to dramatic risk of conflict is the fact that most migration flows do not lead to conflict. Since the 1950s, the majority of countries that have received large numbers of refugees have not engaged in armed conflict [62]. This does not mean that there has not been intergroup conflict, but it does suggest that even a large increase in migration is not necessarily a security risk. Furthermore, scholars such as Michael Humphrey argue that there has been an increased focus in the West on the potential security implications of migration as a result of the terrorist attacks on the United States on 11 September 2001 [63]. Humphrey suggests that this framing of migration, particularly in the United States, can obfuscate the fact that it is a necessary and intrinsic part of global population dynamics, and one that does not usually pose a security threat [63]. 

This underscores the fact that other factors may be more significant than migration in determining whether conflict will occur. For example, sociologist Slettbak argued that populations often unite after natural disasters and that the risk of violent conflict after such an event can be quite low [64]. Slettbak draws from an expansive 1961 study (for which over 16,000 interviews and questionnaires were conducted) about human behavior during disaster situations. The study concluded that most disasters “produce a great increase in social solidarity… [which] reduces the incidence of most forms of personal and social pathology” ([64], p. 165). Slettbak also notes that disasters may even unify those whose differences might initially be viewed as sources of conflict—for example, ethnic, socioeconomic, or religious differences [64]. This suggests that, for example, even if migration heightened ethnic or racial tensions, a natural disaster might not increase risk of conflict but might actually result in a more unified population. 

Another potential stabilizing factor that has been discussed frequently is general political stability and the ability and capacity of government to maintain peace [61,65]. If the state has the capacity to provide resources, such as healthcare, education, and livelihood assistance in the event of economic recession, it has a greater capacity to maintain order and stability in the face of environmental change [16]. A state may mitigate potential conflict by providing livelihood assistance when necessary, furthermore a well-developed and functioning government may have adaptation or mitigation plans in place to deal with the challenges of climate change [16]. In particular, the development of early warning systems could aid in early and informed decision-making, which would allow migration to be successfully employed as an adaptation measure, as opposed to a last resort [66]. 

Thus, despite the added cultural and socioeconomic stress of migration, conflict is not inevitable or even likely in well-established political states. Democracies, in particular, have been shown to be protective against conflict because these states are accountable to their populations and, due to this responsibility, may take steps to conserve water and land in the event of resource depletion [17]. Thus, even with the added stress on resources, a state can perform stabilizing functions to help maintain peace. This directly challenges the neo-Malthusian model, which fails to account for stabilizing forces, such as political and economic stability, that may outweigh competition for resources and thus limit conflict [61]. This echoes the findings that democratic states and institutions have the capacity to regulate environmental degradation and preserve peace [17,67]. Additionally, it has been suggested that human ingenuity and technology have the potential to outweigh the potential dangers of environmental degradation [1]. 

The potential flaws in the neo-Malthusian model have also been challenged empirically Increased resource scarcity and increased population pressure, for example, have only weakly been linked to increased risk of domestic conflict [68]. This suggests that resource scarcity may not have a dramatic impact on risk of conflict, at least domestically. However, current research on this link does not include potential climate projections, which may increase land degradation and resource scarcity on an unprecedented level. Additionally, it is possible that in the context of international conflict the results would be different. However, it is still important to acknowledge that the neo-Malthusian paradigm may not always hold true [68]. 

The fact that migration has been linked to increased conflict in the past does not mean that climate-migration will increase the risk of conflict. The primary challenge of linking climate-migration to conflict is the difficulty in determining the relative importance of climate-migration, among the many other drivers, as a potential factor for conflict. Furthermore, it is important to note that the pathway is non-linear and in some cases climate-induced conflict may in turn cause migration. For example, it has been suggested that the current crisis in Syria (beginning in 2011) was due in part to a period of intense drought and water scarcity [69]. By 2015, an estimated 6 million have been displaced, the majority of whom have left the country [70]. This migration may in turn lead to future conflict, indicating that not only are there complexities associated with the climate-migration-conflict pathway but also that we must consider the three factors as interacting nonlinearly [71]. 

## 4. Considerations for Future Research Directions

At present there appears to be no clear consensus as to how substantial an impact climate change will have on worldwide conflict or the role that migration may play as a part of that pathway. Despite this uncertainty, it is clear that climate change is one of the most significant threats that mankind will need to address in the coming decades, and the potential impacts of climate variability and change on migration and conflict will remain an important area of research and policy planning. 

The major contention in this research surrounds the importance of climate and migration as drivers of conflict compared to other potential factors that may either enhance or suppress risk of conflict [72]. Given the complexity of this issue, future research should seek to understand how climate interacts with other key governance, economic, cultural and social factors. In order to address the interactions of these multiple drivers more thoroughly, future research will be especially valuable to the extent that it can be focused on specific places and contexts. 

It has been suggested that even focusing on a regional scale is too broad due to the individual governmental, economic, social, and environmental factors that play key roles in this setting and which may vary dramatically by region or country [73]. Local variation has been inadequately addressed to-date in most research on this topic [73]. In the remaining sections, we highlight five questions that can help to prioritize and guide future research at the local level: What are the local climate risks? What is the potential for resource scarcity? Is migration economically viable? What is the status of local stabilizing or destabilizing factors? and Is there a local history of conflict? 

### 4.1. What Are the Local Climate Risks?

Climate risks vary greatly at the local level, whether related to extreme events or to long-term climate change. Understanding the local context of climate variability and change is essential to understanding the role of climate as a driver of migration and/or conflict. This highlights the importance of both highly resolved local historical weather as well as climate projection data. Given that climate change will be superimposed on pre-existing local conditions, previous weather trends will influence the impact that climate change has on a given population. For example, in hot cities, high temperatures are associated with fewer health impacts compared to cooler cities [74]. This highlights the importance of understanding both climate projections and local historical weather when assessing climate vulnerability. This can be applied to climate-migration, as well. For example, the Rhine delta in the Netherlands experiences annual flooding and strategies for flood-risk management have been practiced for thousands of years [75]. Sea-level rise poses a serious threat to the river basin, but a history of flooding and a longstanding commitment to “living with the floods” has catalyzed Dutch preparedness plans [75]. In this way, the ways in which a community has adapted to deal with historical weather conditions can impact its resilience, which may ultimately influence migration due to climate change. 

### 4.2. What Is the Potential for Resource Scarcity?

Climate change will restrict resource availability in many areas of the world [76]. In some regions, stabilizing factors such as governments with adaption plans or economic safety nets will be able to reduce the potential for conflict. However, in certain areas, resource scarcity may be too severe to overcome without negative consequences. The Middle East and North Africa, described as the most “water-scarce region of the world”, have seen significant increases in droughts over the last two decades [69]. In this area, decreased water availability has significant consequences as some Arab countries are dropping below levels of absolute scarcity per capita [69]. In these contexts, it is possible that increased water scarcity is more likely to cause conflict than in moister regions. Furthermore, it is important to note that resource scarcity is not isolated from political stability. Regions that have historically been dealing with issues of resource scarcity may have weaker or less developed governments and institutions [76]. Thus, regions already experiencing resource scarcity may be doubly vulnerable to conflict as a result of climate-migration because of a pre-existing lack of resources, but also because of a potential lack of mediating forces such as political stability.

Resource scarcity is also important to consider in terms of agriculture. Specifically, whether or not the economy is dependent on the land is a crucial factor for determining how significant environmental stress will be as a driver for migration. Countries that may depend heavily on agriculture and would be devastated by major failures in crop are particularly at-risk to the negative impacts of climate change [77]. A number of studies have been conducted on the potential impact of climate change on agriculture, particularly in developing countries. These often suggest that there could be substantial loss to agricultural productivity over the next century in many regions, particularly in developing countries [77,78,79,80].

Agriculturally based developing countries are of particular concern, as they may also have poor infrastructure and low levels of technology to respond to climate risks [79]. Adaptation to better prepare for the impact of climate change on global food systems has been called for since the 1990s [80]. However, a 1994 study that simulated adaptive measures to combat the impact of climate change on farming found that farm-level adaptations may be unable to mitigate the negative impacts on cereal yields in developing countries [80]. Because adaptation may not be feasible, particularly in developing countries, climate change may act as a significant factor in causing migration out of these areas.

### 4.3. Is Migration Economically Viable?

Economic development can both contribute to and limit migration. In poorer regions, migration can be used to diversify income, and may be a necessary strategy for survival after extreme events. Alternatively, severe poverty, which is associated with limited resources, may limit capacity for migration [81]. Income, which is often used as a proxy for poverty, has been found to have different impacts on migration choice after natural disasters: some research has found an inverted U-shaped pattern where the middle incomes migrate more frequently and the low and high incomes more often stay [81,82]. However, others have not been able to replicate this migration pattern [82]. Again, this factor cannot be isolated from its lateral counterparts. As Massey reminds us, the source of income is of particular importance [38]. As a result, we should consider economic levels in conjunction with the local economy to understand the relative weight of these factors [38]. 

This applies to slow-onset climate change, as well. Sea-level rise, for example, would seem to result in inevitable displacement. However, in some cases, radical adaptations are being proposed that would prevent the need for migration. For example, the island of Kiribati is facing imminent sea-level rise and a decreasing freshwater lens due to saline intrusion [83]. However, despite these risks, the island is considering dramatic alternatives to migration, including building a $1 billion sea-wall and/or a $2 billion dollar floating island [84]. These adaptations are being considered because of lack of a viable migration strategy [84]. This is due in large part to the cultural and psychological implications of abandoning Kiribati, as well as the extreme poverty of the islanders who are unable to afford to move even if willing [84]. 

### 4.4. What is the Status of Local Stabilizing or Destabilizing Factors?

Gleditsch stressed the importance of creating better indicators to measure the role of political institutions in conflict research almost 20 years ago [85]. However, there are limited studies that successfully incorporate these stabilizing forces. One notable exception is Hummel’s (2015) thorough review of climate change and migration in Mali and Senegal, which placed particular emphasis on political and legal frameworks that influence migration [86]. Hummel found that in the West African Sahel, while environmental stressors can influence mobility, the environment is not usually the most important factor that causes people to migrate [86]. Additionally, Hummel suggests that because of the cultural history of migration of the people of West Africa, political strategies should not attempt to limit migration, but instead should try to harness it as a tool for adaptation. This demonstrates the capacity of political institutions to facilitate adaption and reduce vulnerability. This can also be done through the development of infrastructure to help populations withstand extreme events [87]. Physical infrastructure, such as irrigation systems, well-maintained buildings and roads, as well as healthcare infrastructure can greatly reduce susceptibility [87]. In contrast, vulnerability is increased in regions with political insecurity and in those that lack adequate infrastructure and services provided by the government [87].

Furthermore, van der Land and Hummel (2015) conducted a study that focused on the role of education in environmentally induced migration [88]. They found that in Mali and Senegal, formal education reduced vulnerability to environmental stressors and as a result reduced migration for economic reasons [88]. They attribute this finding to education making individuals less reliant on environmentally sensitive sources of income [88]. In contrast, those with lower levels of formal education are substantially more likely to migrate than their counterparts [88]. 

There are a number of other stabilizing forces that may lessen the potential for conflict. For example, increased social capital has been linked to increased ability to withstand natural disasters, particularly in rural areas [89]. Neighborhood networks and cohesion can be utilized during extreme events as ways to provide and receive information and assistance [89]. This type of social capital has also been incorporated into effective natural disaster preparedness planning [90]. Preparedness has the potential to reduce negative impacts from climate events thus reducing the risk of conflict. In contrast, social isolation is considered to greatly increase vulnerability by limiting the capacity of individuals to draw on social networks for support during and after natural disasters [90]. 

Technology can also be used to support resiliency. For example, farmers can use different technologies or combinations of technologies to adapt to climate change and prevent resource depletion [91]. In addition, new cultivars are being developed that will be more resilient to climatic changes, such as drought [92]. This technology may also help moderate resource scarcity thereby reducing vulnerability. Limiting the impacts of climate change on a given area may reduce the need for migration. Additionally, limiting these impacts in areas that are receiving migrants may limit the risk of conflict. 

### 4.5. Is There a Local History of Conflict?

A region that has a history of tension or conflict (particularly one that is related to migration) might be susceptible to increased instability due to migration influx. For example, Bangladesh and India have a history of tension since the Indian Partition in 1947. The partition resulted in the split between India and Pakistan (which would later divide into Pakistan and Bangladesh). Modern tension in this region has been driven in large part by migration from Bangladesh into India [93]. There has been growing concern about increasing numbers of environmentally driven migrants into India over the last 20 decades [94]. Much of this migration is due to river flooding and erosion, which have had negative impacts on Bangladeshi agriculture and economics [95]. Many of those migrating out of Bangladesh sought a better life in India. However, should increased coastal flooding strain West Bengal as well, Bangladeshi migrants may be diverted elsewhere or put in direct conflict for environmental resources, employment, and physical space with Bengali residents [96]. This region demonstrates an area that has already seen conflict over migration and as a result might be more at risk of future conflict should climate change lead to increased rates of migration. 

## 5. Conclusions

Our review of the literature on the climate-migration-conflict nexus suggests that scholars generally agree that climate change has the *potential* to lead to increased migration and increased risk of conflict. However, where the literature diverges is in the relative importance of climate and migration as drivers of conflict, compared to other factors. As this field has developed, there has been increasing recognition of the complexity of the systems linking climate, migration and conflict, and the extent to which this system depends on social, demographic, economic, and political drivers that interact with climate variability and change. All of these are very location-dependent. Thus, future research can help to inform our understanding of the contexts in which climate might increase risk of conflict by focusing on the local interplay of these multiple drivers. It may be unrealistic to expect that a general theory will emerge any time soon that can predict where climate-related migration and conflict is likely to occur in the future. However, it is hoped that place-based research will proceed that can inform preparedness plans and policy decisions that will be essential to mitigate the potential health impacts of climate change, migration, and conflict. 

## Figures and Tables

**Table 1:** Examples of climate extremes leading to migration and conflict since 2000. Despite the increasing body of literature on the theory and potential for climate related migration to result in conflict, there are surprisingly few case studies on recent climate extremes that lead to migration and conflict (see Reuveny, 2007 for a meta-analysis of historical examples) [1]. We have selected a number of key examples that are representative of the current literature.

Type of Climate Event	Region/Country	Time Period *	Major Impacts on Migration	Presence of Conflict	Sources
Drought/soil degradation	Kenya	2004 & 2007	Increase in temporary labor migration with decreasing soil quality	n/a	Gray, 2011 [2]
Heat stress	Pakistan	1991–2012 **	Increase in long-term migration of men	n/a	Mueller *et al.*, 2014 [3]
Forest fires	United States	2010	Increased intention to migrate	n/a	Nawrotzki *et al.*, 2014 [4]
Flooding/Cyclone	Bangladesh	2009	Increase in male rural-urban migration	Moderate evidence of contributing to intra-familial conflict	Mallick & Vogt, 2012 [5]
Flooding	Pakistan	2011–2012	Increase in rural-urban migration	Strong evidence of violent conflict over political power between migrants and non-migrants	Bhattacharyya, & Werz, 2012 [6]
Drought	Syria	2006–2014	Increase in rural-urban migration	Moderate evidence of contributing to violent conflict	Gleick, 2014 [7]
Drought/water scarcity	Western Sahel	2005–present	Increase in labor-related migration of pastoralists	Strong evidence of contributing to clashes between pastoralists and farmers over resources	UNEP, 2011 [8]; Nyong, 2011 [9]
Droughts	Peru & Bolivia	1996–present **	Increase in labor-related migration of farmers due to increasingly devastating droughts	Strong evidence of contributing to conflict between farmers over resources and ethnic conflict between farmers and indigenous migrants	Hoffman & Grigera, 2013 [10]; Carrol & Schipani, 2011 [11]
Desertification	Nigeria	~1993–2013 **	Increase in labor-related migration of farmers due to increasing desertification	Strong evidence of ethnic conflict between farmers over rangeland	Folami, 2013 [12]; Werz & Conley, 2012 [13]

* Time periods are approximate. Ranges are based on data collection time periods when concrete dates were not provided for the climate extreme itself. ** These studies have data collection preceding 2000 but migration and/or conflict occurring after 2000 and as such were included.
